# **Training, Migration and Retention of Doctors: Is Ireland a Danaides’ Jar?** Comment on "Doctor Retention: A Cross-sectional Study of How Ireland Has Been Losing the Battle"

**DOI:** 10.34172/ijhpm.2020.217

**Published:** 2020-11-01

**Authors:** Guillaume Chevillard

**Affiliations:** Institute for Research and Information in Health Economics, Paris, France.

**Keywords:** Workforce, Doctor Retention, Migration, WHO Global Code, Ireland, Training

## Abstract

In a context of global shortage of doctors, Ireland is in a paradoxical situation: the country trained a lot of medical students, native or foreign, but has difficulties to retain them. The paper of Brugha and his colleagues analyzes junior doctors’ migration intentions, the reasons they leave, the likelihood of them returning and the characteristics of those who plan to emigrate. Results show determinants of junior doctor’s emigration and may be useful to better calibrate the doctors’ retention strategy of Ireland.

Brugha and colleagues^1^ aim at measuring junior doctors’ migration intentions in Ireland, the reasons they leave, the likelihood of them returning and the characteristics of those who plan to emigrate. The authors used an online survey of junior hospital doctors and cross their career intentions (remain in Ireland, leave but return later, leave and not return and to quit medicine) with several individual characteristics (demographic status, nationality, level and type of study, specialty, and the intended country of migration if leaving). The study answers to the need of evaluate a public policy deployed 3 years previously and aiming at improving graduate retention in the public health system and thus limit the exit flows of doctors. This is a major stake in Ireland where the health coverage is particularly difficult to reach.

 Main results show first that very few respondents intend to quit medicine (3%). On the other hand, 17% of respondents plan to leave Ireland and not return and 34% plan to leave Ireland but return later. Finally, just under half of the respondents (45%) intend to remain in Ireland. These results already confirm the first trends described by the authors in the context about the low retention of doctors trained in Ireland, and highlight the emergency of the situation. The authors show then typical profiles of doctors according to their career intentions, with interesting details, for instance, about leavers or returners. All of these results are operational and can help improve doctor’s retention strategies of Ireland, of course, but also for other countries experiencing doctor’s emigration.

 This survey shows, and it is of main interest here, that the retention strategy did not address the root causes of the unsatisfactory training and working conditions that are driving doctors emigration. The national retention strategy seems to only have improved a few parts of the education and training stages. Some training dimensions with the greatest potential of improvement depend on external financial constraints that the retention strategy cannot address. For instance, there is a need of a sufficient number of consultant trainers, but the austerity policy started in 2012 leads to a decrease of their number. Finally, this study highlights the need to use several levers to improve retention of doctors, and it also involves bringing back the doctors who trained abroad for their specialty by facilitating career paths. Taking into account all these results and recommendations will perhaps reduce the emigration of doctors in Ireland and avoid continuing to train doctors who would go away like the water poured by the Danaides in a jar with holes.

The paper of Brugha et al is a major interest in a context of international migration of doctors that questions the balance between human resources in health and the healthcare needs of populations. Two contexts elements are important to know when analyzing migration of doctors. First, there is a global shortage of doctors and it could reach 400000 doctors in 2030 for Organisation for Economic Co-operation and Development (OECD) countries^2^ and 2.5 million for the low incomes countries.^3^ Second, most of countries have an unequal geographic distribution of doctors leading to medically underserved areas where there is a local shortage of doctors. In such areas, inhabitants have more difficulties to access to healthcare.^4^

Publics authorities of many countries deployed several policies in order to answer these two types of shortage, by reaching universal health coverage^5^ and/or attracting and retaining doctors in medically underserved areas.^4^ According to Sousa and her colleagues, three types of policies exist: those on production (eg, increase number of students trained, teaching staff), those who address inflows and outflows (eg, migration and emigration or attract unemployed health workers) and those who address maldistribution and inefficiency (eg, to retain health workers in medically underserved areas). Thus, immigration and emigration of doctors supply in several ways the global stock of doctors, and his evolution, in a country when other flows also impact the stock such as the retirement of doctors or a temporary cessation of activity.^6^ The reasons of doctor’s emigration are multiple and specific to each doctors but the main factors identified are the search for better incomes, better working conditions or the need for training.^7^ Achieving a level of sufficiency in doctors thus requires controlling several of the levers that we have mentioned. Another challenge will also be to retain the doctors both at country level and in areas or specialties with the highest needs.

According to a recent OECD report,^8^ international migrations of doctors increase of 50% between 2006 and 2016 and there are now 500000 foreign-trained doctors in OECD countries. The main destinations are the United States, the United-Kingdom and Germany, when another survey^9^ shows that the main supplier countries are India, Philippines, Canada and the United Kingdom. Finally, in Europe, there is also an increase of doctors migration through European Union (EU) members, especially since the directive of 2005 on the recognition of professional qualifications and the and the gradual enlargement of EU.^7^

In such context, the Irish situation, highlighted in the paper, appears very interesting and paradoxical in some case. First, there is a global shortage of doctors in Ireland while the ratio of local trained-doctors is over the OECD average, illustrating the already documented doctors’ emigration.^10^ Also it is a country which trains a lot of foreign students, without being able to keep all of them. This is partly due to the fact that a preference is given to local students in the choice of their internship position, and that the arrival of foreign students is a means of financing the training of doctors^1^. Almost half of the students are foreigners from Malaysia or Canada, and in most cases, they do not pursue their postgraduate training. When at the same time a significant part of the trained doctors, Irish or not, emigrate. In the end, Ireland faces doctor’s shortage and still has to recruit foreign-trained doctors: they increased by 50% between 2011 and 2017 where they now represent 42% of Irish doctor’s workforce.

 The paper of Brugha et al, through the example of Ireland, clearly shows the complexity of achieving doctor’s coverage in a country and the need to operate on several levers. We have seen that increasing the number of students alone is not enough to reach it, in particular because of incoming and outgoing flows of doctors illustrating very heterogeneous behaviours and individual strategies.

 The Irish example can also be interesting for other countries which train a lot of foreign students and face doctor’s retention issues. From a European perspective, the comparison with the Romanian example can be very instructive and it could be interesting to replicate this research to see if the factors explaining career intentions are similar or not. Finally, as the authors point out at the end, the recent health crisis has been the reason for doctors’ returns. It will also be interesting to study if these returns are temporary or if they will last and if so, what are the reasons.

## Ethical issues

 Not applicable.

## Competing interests

 Author declares that he has no competing interests.

## Author’s contribution

 GC is the single author of the paper.

## References

[1] Brugha R, Clarke N, Hendrick L, Sweeney J (2020). Doctor retention: a cross-sectional study of how Ireland has been losing the battle. Int J Health Policy Manag.

[2] Scheffler RM, Arnold DR (2019). Projecting shortages and surpluses of doctors and nurses in the OECD: what looms ahead. Health Econ Policy Law.

[3] World Health Organization (WHO). Global Strategy on Human Resources for Health: Workforce 2030. Geneva: WHO; 2016.

[4] Ono T, Schoenstein M, Buchan J. Geographic Imbalances in Doctor Supply and Policy Responses. Paris: OECD; 2014.

[5] Sousa A, Scheffler RM, Nyoni J, Boerma T (2013). A comprehensive health labour market framework for universal health coverage. Bull World Health Organ.

[6] Organisation for Economic Co-operation and Development (OECD). The Looming Crisis in the Health Workforce: How Can OECD Countries Respond? Paris: OECD; 2008.

[7] Ognyanova D, Maier CB, Wismar M, Girasek E, Busse R (2012). Mobility of health professionals pre and post 2004 and 2007 EU enlargements: evidence from the EU project PROMeTHEUS. Health Policy.

[8] Organisation for Economic Co-operation and Development (OECD). Recent Trends in International Migration of Doctors, Nurses and Medical Students. Paris: OECD; 2019.

[9] Moullan Y. The International Migration of Doctors: Impacts and Political Implications. Issues in Health Economics; 2014.

[10] Clarke N, Crowe S, Humphries N (2017). Factors influencing trainee doctor emigration in a high income country: a mixed methods study. Hum Resour Health.

